# Anti-epileptics and pregnancy: an update

**DOI:** 10.1007/s00415-018-9058-6

**Published:** 2018-09-20

**Authors:** A. Al-Ansari, N. P. Robertson

**Affiliations:** 1 Department of Neurology, University Hospital of Wales, Heath Park, Cardiff, CF14 4XN UK; 2 Department of Neurology, Institute of Psychological Medicine and Clinical Neurosciences, Cardiff University, Cardiff, CF14 4XW UK

In utero exposure to selected anti-epileptic drugs (AEDs) has an established association with adverse outcomes in exposed offspring. However, since the majority of women with epilepsy still require on-going treatment with AEDs during pregnancy, quantifying the risk of related adverse events as well as identifying potential modulating factors remain highly relevant. Although the teratogenic effects of valproate are now widely recognised, there remains a need to clarify risks of long-term intellectual impairment in children exposed to AEDs in general and to understand any underlying mechanisms of action.

This month’s journal club reviews three papers which explore the use of anti-epileptic drugs in pregnancy. The first is a large, national, population-based study exploring association between AED exposure and performance in language and mathematics tests in school-aged children. The second examines the relationship between folic acid levels and verbal ability in offspring of mothers with epilepsy and the third provides an insight into potential biological mechanisms by considering the action of valproate on the placenta.

## Association between prenatal valproate exposure and performance on standardized language and mathematics tests in school-aged children

In this study, the core data were provided by Statistics Denmark which then allowed linkage across a selection of national registers, and for information on several domains to be collated on a large number of children. All live births in Denmark between 1997 and 2006 were identified. Children with missing information on performance in national tests or other covariates were excluded. Medical exposure to AEDs was defined as 30 days from the first day of the last menstrual cycle to 1 day before birth. The main outcome of the study was performance in National tests in Danish and mathematics. The results of all tests completed between 2010 and 2014 were presented as standard deviations from the mean (*z* scores) for comparison. Scores were adjusted for covariates including sex, calendar year, mother’s educational level and household income.

A total of 479,027 children were included, of whom 253 were exposed to sodium valproate as monotherapy, 396 to lamotrigine, 294 to carbamazepine, 236 oxcarbazepine, 86 phenobarbital and 188 to clonazepam. Children exposed to valproate monotherapy performed significantly worse than AED-unexposed children, even when outcomes were adjusted for socioeconomic factors. However, there was no significant difference in the test results of children exposed to lamotrigine or alternative AEDs when compared to unexposed children.

*Comment.* Strengths of this study include the large study population, access to accurate and well-curated datasets from the Danish registry system and the ability to compare standardized test results over a 4-year period. The authors concede that missing information regarding folic acid use is a limitation, and also point out that children attending private schools were not included in the study, as they did not participate in national tests. Further limitations include the difficulty of adequate adjustment for the complex socioeconomic covariates which influence a child’s school performance.

Despite these issues, this is a large and robust study which supports previous observations of the association of prenatal valproate exposure with poorer school performance, but does not see these adverse effects in a range of alternative AEDs and also supports the use of lamotrigine as a safer alternative.

Skou Elkjaer et al. (2018) JAMA Neurology 75(6):633–671.

## Verbal abilities in children of mothers with epilepsy: association to maternal folate status

Folate is a type of vitamin B whose deficiency has been associated with neural tube defects. Many AEDs interact with folate metabolism and as a result women with epilepsy are advised to take folic acid supplements during pregnancy. Recent research has also linked folate deficiency with autism and lower intellectual ability in offspring.

This prospective, epidemiological study investigated the effect of maternal folic acid supplementation, maternal plasma folate levels and anti-epileptic drug concentrations on verbal ability in AED-exposed children. The study population consisted of participants in the Norwegian Mother and Child Cohort Study which is an on-going population-based study conducted by the Institute of Public Health. Parent-completed questionnaires on background, medical history, folic acid intake and child development were completed at 17–19 weeks’ gestation, 30 weeks’ gestation and at age 18 and 36 months. Blood samples were also collected from the mother at weeks 17–19 of pregnancy and from the umbilical cord immediately after delivery. Covariates included parental higher education, total household income, unplanned pregnancy, smoking and alcohol intake. Data were analysed from three groups: AED-exposed children born to mothers with epilepsy, AED-unexposed children born to mothers with epilepsy and children of mothers without epilepsy. Groups were further sub-divided according to folic acid supplementation.

In the AED-exposed group without folate supplementation 34% and 24% had global language delay at 18 months and 36 months, respectively, compared to 11% and 6% in the control group without epilepsy. In the AED-exposed children of mothers with epilepsy who had taken folic acid, 17% had global language delay at 18 months. Furthermore, the number of lamotrigine-exposed children with language delay was higher in the group without folic acid supplementation compared to the group that had received supplements. Higher maternal plasma valproate concentration correlated significantly with a lower global language score at age 18 months. However, no correlation between folate dose or concentration and verbal ability was identified.

*Comment.* This study involves a large dataset, effective validation of maternal diagnosis of epilepsy and appropriate attempts to adjust for confounding factors. However, there are some limitations which include potential sub-optimal timing of blood tests for folate and AED levels at 17–19 weeks’ gestation rather than the first trimester. Nevertheless, this study convincingly demonstrates a protective effect of folic acid supplementation on language development in AED-exposed children. This clinically relevant observation is in keeping with previous research; however, more information on the effect of folate supplementation on children exposed to lamotrigine would be informative.

Husebye et al. (2018) Neurology 91(9):e811–e821.

## Adverse placental effects of valproic acid: studies in perfused human placentas

This paper explores the effect of short exposure to valproic acid on placental expression of transporters for compounds essential for foetal development. Placentas were obtained from term caesarean deliveries of women without a known diagnosis of epilepsy. Cotyledons were cannulated and perfused in the absence or presence of valproic acid at therapeutic (42 µg/ml and 83 µg/ml; *n* = 6/group) and supratherapeutic (166 µg/ml; *n* = 6) concentrations for 180 min. Expression of genes for various transporters was analysed in the cotyledons after this time period.

The study demonstrated that exposure to valproic acid results in significant down-regulation of gene expression of important placental transporters, in particular, FOLR1 which encodes the folate receptor alpha and SLC2A1 and GLUT1 which encode glucose carriers. Folate concentrations within placental tissue exposed to valproic acid were reduced by 25–35% compared to the control group, although did not reach significance (*p* = 0.059). Up-regulation of the SLC6A4 gene which encodes the serotonin receptor was also observed and may be of relevance as high levels of serotonin have previously been linked to autism.

*Comment.* This study is one of the few to explore the effects of sodium valproate on the human placenta. The study was designed to imitate in utero conditions including pH and oxygen tensions. Limitations include the use of placentas collected at term and for a short time interval of 180 min. It has been suggested that the adverse effects of valproic acid are more likely to take place in early pregnancy within a more complex biological setting and over a considerably longer time period.

Rubinchik-Stern et al. (2018) Epilepsia 59:993–1003.

*Conclusion.* The results of these studies support recent evidence that valproate is associated with poorer educational attainment and reiterates the importance of peri-conceptual folic acid supplementation. Whilst the first paper has proposed lamotrigine as a safer drug in terms of long-term school performance, the second study found an association between lamotrigine-exposed children of mothers who had not taken peri-conceptual folic acid and language delay. With the increasing availability of newer AEDs, including levetiracetam, and their preferential use in pregnancy above some of the traditional agents, further research to establish data of adverse events in offspring of mothers with epilepsy is needed.

